# A randomized controlled trial of a smoking cessation intervention conducted among prisoners

**DOI:** 10.1111/add.12084

**Published:** 2013-03-11

**Authors:** Robyn Richmond, Devon Indig, Tony Butler, Kay Wilhelm, Vicki Archer, Alex Wodak

**Affiliations:** 1 School of Public Health and Community Medicine, UNSWSydney, NSW, Australia; 2 Centre for Health Research in Criminal Justice, Justice Health NSWEastgardens, NSW, Australia; 3 National Centre in HIV Epidemiology and Clinical Research, University of New South WalesSydney, NSW, Australia; 4 Faces in the Street, Urban Mental Health Research Institute, St. Vincent's HospitalDarlinghurst, NSW, Australia; 5 School of Psychiatry, UNSWSydney, NSW, Australia; 6 Alcohol and Drug Service, St. Vincent's HospitalDarlinghurst, NSW, Australia

**Keywords:** Cognitive therapy, nicotine dependence, nicotine patch, nortriptyline, prisoner, smoking cessation

## Abstract

**Aim:**

To evaluate the efficacy of nortriptyline (NOR) added to a multi-component smoking cessation intervention, which included cognitive–behavioural therapy (CBT) and provision of nicotine replacement therapy (NRT).

**Design:**

Randomized controlled trial (RCT) comparing two study groups with blinded follow-up at 3, 6 and 12 months. Both groups received a multi-component smoking cessation intervention comprising two half-hour individual sessions of CBT and NRT with either active NOR or placebo.

**Setting:**

Prisons in New South Wales (17) and Queensland (one), Australia.

**Participants:**

A total of 425 male prisoners met inclusion criteria and were allocated to either treatment (*n* = 206) or control group (*n* = 219).

**Measurements:**

Primary end-points at 3, 6 and 12 months were continuous abstinence, point prevalence abstinence and reporting a 50% reduction in smoking. Smoking status was confirmed by expired carbon monoxide, using a cut-point of ≤10 parts per million.

**Findings:**

Participants' demographics and baseline tobacco use were similar in treatment and control groups. Based on an intention-to-treat analysis, continuous abstinence between the treatment and control groups was not significantly different at 3 months (23.8 versus 16.4%), 6 months (17.5 versus 12.3%) and 12 months (11.7 versus 11.9%).

**Conclusion:**

Adding nortriptyline to a smoking cessation treatment package consisting of behavioural support and nicotine replacement therapy does not appear to improve long-term abstinence rates in male prisoners.

## Introduction

The prevalence of smoking in the general community in Australia decreased from 28% in 1998 to 20% in 2010 among men and from 22 to 16% among women 1. While tobacco control strategies have decreased tobacco use in the Australian general population, smoking rates remain high in disadvantaged populations 2,3 such as prisoners (85%), people with a mental illness (50–80%), Aboriginal Australians (48%) and illicit drug users (71%) 2,4–9.

Prisoners endure some of the worst health outcomes of any identifiable population group in the community. They are characterized by social, health and psychological disadvantage, poor educational attainment, unemployment, social isolation, interpersonal conflicts, Aboriginal heritage and alcohol and drug problems 7,8.

Surveys of prisoners in New South Wales (NSW), Queensland (QLD) and Victoria (VIC) found high levels of physical and mental ill health 7–11. Depression is known to be associated with higher rates of smoking 12 and smoking cessation can trigger depression, thus diminishing the effectiveness of cessation interventions in populations with high background levels of depression 13. Prisoners use mainly ‘roll-your-own’ tobacco, with higher nicotine and tar content than manufactured cigarettes 7. Aboriginal people represent approximately 2% of the general population, but are as high as 21% in NSW and 30% in Queensland prison populations 14.

Therefore, not only do prisoners have extremely high rates of smoking, but most come from populations with high smoking rates in Australia: Aboriginal people, people with mental illnesses and people with severe alcohol and drug problems. However, prisoners are health conscious: three-quarters wish to quit smoking 7,8, 66% had exercised ≥30 minutes per day for the previous 4 weeks and 62% are concerned about the nutritional value of prison food 7,8. Prison provides an opportunity for inmates to improve health, including smoking cessation.

Several studies have described the smoking and demographic characteristics of current prisoners in Australia 3,7,8,10,15, the United States 16,17, United Kingdom 18,19 and Poland 20. Some before–after studies evaluated smoking cessation interventions among prisoners 3,21–25. To our knowledge, only one prison-based randomized controlled trial (RCT) has been published on smoking cessation. Cropsey *et al*. 16,26,27 compared an intervention and waiting-list control group (who received treatment after 6 months) among female prisoners in a prison in southern United States. The multi-component intervention included smoking cessation treatments recommended by Cochrane and Australian guidelines 28–32. Studies conducted among US smokers reported benefits from adding nortriptyline (NOR) to a multi-component intervention 33–35.

Our study aims to evaluate the efficacy of adding NOR to a multi-component intervention involving brief cognitive behavioural therapy and nicotine transdermal patch (NRT) among male prisoners.

## Methods

### Study design

Prisoners providing written informed consent were assessed at pre-treatment, 3, 6 and 12 months following initial assessment. Participants were recruited by referral from clinic staff, flyers and widely distributed posters.

Eligibility criteria were: male; aged more than 18 years; incarcerated for ≥1 month with ≥6 months of the current sentence remaining; English speaker; score of ≥5 on the Fagerström Test for Nicotine Dependence (FTND) (indicating moderate/high nicotine dependence) and readiness to quit 36,37. Participants were required to provide contact details of family or friends to improve community follow-up following release. Exclusion criteria were: female, current significant cardiovascular or mental illness (major depressive disorder, bipolar disorder; threats of suicide or repeated deliberate self-harm; current psychotic disorder); current use of antidepressant or antipsychotic medication; use of monoamine oxidase inhibitors within 2 weeks; known allergies to the study drugs and a life-threatening illness.

We accepted prisoners with mild to moderate depression or a past depression history. However, we excluded people with current major depression (as they were likely to be already taking antidepressant medication and we were concerned about the potential for drug interaction and increased side effects, including risk of suicide).

Current major depression was identified by the prison doctor conducting medical assessments of all potential participants, by psychiatrists or ascertained from a prisoner's medical record.

Inmates meeting inclusion criteria were allocated randomly to one of two study groups using a randomization algorithm. The treatment condition included brief cognitive–behavioural therapy (CBT), active NOR, active transdermal patch, a booklet to assist prisoners at times of stress, a quit calendar developed by prisoners in the pilot trial and access to the Quitline telephone counselling service (provided to the community by the NSW Health Department). The control condition was the same, but included placebo NOR.

### Setting and sample

We recruited 425 participants from 17 prisons in NSW and one prison in Queensland between August 2006 and September 2009. A further nine NSW prisons participated in the follow-up assessment as prisoners were transferred to other facilities. This sample size was required to detect a 14% difference with a power of 0.8 and significance level of 0.05. We recruited 20% more subjects to allow for dropouts. This target was based on the only study investigating the use of brief CBT, NRT and NOR 38,39.

### Screening assessment to join the study

Initially, the research nurses reviewed the potential participant's medical files applying the exclusion criteria. Those meeting the criteria were screened for medical suitability by a general practitioner (GP) using the screening checklist. If the GP was uncertain about an inmate's health status, they contacted one of the investigators (KW, a psychiatrist; or AW, a physician). GPs prescribed the medication. Compliance was monitored using the prisoner's medical record. All medications were administered and supervised by a nurse at the prison clinic who signed the treatment sheet for each dose.

### Interventions

Table 1 presents the study procedure for the treatment and control groups. The multi-component intervention was identical in each study group, except that the treatment group received the active NOR and the control group received the placebo.

**Table 1:** Study procedure for the intervention and study group

Week	Procedure	Treatment group	Control group	NRT
	Recruitment			
−2	Baseline screening			
−1	Randomization			
1–2	Begin NOR or placebo	Tapered dose^1^		
3	Quit week	✓	✓	✓
4–11		✓	✓	✓
12	First follow-up date	✓	✓	✓
13		Tapered dose^2^		
26	Second follow-up date			
52	Final follow-up			

1 Dosage is 25 mg/day (one tablet) for 3 days and then 50 mg/day (two tablets) for 4 days.

2 Dosage is 50 mg/day (two tablets) for 4 days then 25 mg/day (one tablet) for 3 days. NOR: nortriptyline; NRT: nicotine replacement therapy.

The smoking cessation date was set as the third week following the commencement of NOR (or placebo) treatment to coincide with commencement of NRT. During the 10-week course of patch therapy, a structured tapering system was employed: 21 mg of nicotine per day for the first 6 weeks, followed by 14 mg/day over the next 2 weeks and 7 mg/day in the final 2 weeks. All participants received the active nicotine patch. The treatment group received active NOR while the comparison group received placebo NOR. NOR and NRT were provided to subjects on a daily basis. A medication chart was completed daily to document medication adherence. Participants were instructed on the correct use of the medications, including emphasis on not smoking when using NRT.

#### NOR

NOR was chosen over bupropion as it is less expensive and is also used in Australia as an antidepressant. It is appropriate to use in the prison system as it needs to be administered only once daily. Subjects commenced medication (active or placebo) 2 weeks prior to their quit date to ensure that therapeutic levels of NOR were reached. Subsequent therapy lasted a further 10 weeks. Inmates received NOR 25 mg/day for 3 days, then 50 mg/day for 4 days and 75 mg/day for the remaining 11 weeks. After this, the dose dropped to 50 mg/day for 4 days, then 25 mg/day for 3 days before the NOR was discontinued. The treatment schedule was based on Prochaska *et al*. 38. Active NOR and placebo were provided in identical tablet form. All medications were dispensed daily by nurses at the prison clinic.

#### Brief CBT (bCBT)

Subjects received two face-to-face bCBT sessions delivered by a counsellor in weeks 3 (the quit week) and 5–6. Sessions lasted 30 minutes. Subjects received a booklet developed for this study (by KW) containing strategies to assist coping with stressors such as prison transfer and court appearances and a quit calendar 40.

#### Quitline

Quitline is a telephone counselling service provided to all Australians in the community. Telephone counselling services have been shown to be effective in helping smokers to quit in Australia 41,42. Quitline offers access to self-help resources, advice, support and telephone counselling for inmates wanting to quit. As part of the development of this study, we negotiated successfully with Quitline and the Department of Corrective Services to allow prisoners in NSW access to this service free of charge. Prior to this, Quitline was not an approved number for prisoners to ring.

#### Transfers between facilities and follow-up

Anecdotal reports suggest more than 100 000 prisoner movements (involving the approximately 9500 prison inmates in NSW) every year. Arrangements were made to transfer medication with the participant when these movements occurred. Follow-up assessments were conducted at 3, 6 and 12 months post-treatment by a prison nurse research assistant who was blind to group allocation.

### Measures

#### Smoking outcome measures

##### Outcomes

The primary outcome measures were continuous abstinence and point prevalence abstinence at 3, 6 and 12 months. Continuous abstinence is defined as abstinence between quit day and a specified follow-up period (in our case 3, 6 and 12 months) 39. Point prevalence abstinence is defined as the proportion of subjects who have not smoked during a particular period 39. Smoking reduction was based on a self-assessment of whether participants had reduced their daily consumption of cigarettes by 50% or greater (including abstinence) relative to baseline 43.

Outcomes were determined on an intention-to-treat basis 44. That is, participants who missed a follow-up assessment were regarded as smokers. At 3, 6 and 12 months, subjects who reported any smoking whatsoever, or whose expired carbon monoxide (CO) levels were 10 parts per million (p.p.m.) or over, were classified as continuing smokers. Current abstinence from smoking was confirmed using a Micro II Smokerlyser (Bedfont Scientific Ltd, Kent, UK), which assesses breath CO levels. A CO level of <10 p.p.m. indicated that the subject had probably not smoked in the previous 8 hours.

#### Measures of nicotine dependence, psychopathology and quality of life

Smoking history questions assessed the number of cigarettes smoked before imprisonment, years of regular smoking and prior quit attempts. Readiness and motivation to quit smoking was also assessed using the Crittenden criteria 37. Nicotine dependence was assessed using the FTND 36, which measures smoking behaviours to determine physical dependence on a scale from 0 to 10. Scores of 6 and above indicate ‘moderate’ to ‘high’ nicotine dependence 36. The Minnesota Nicotine Withdrawal Questionnaire 45,46 measures craving for cigarettes, irritability, frustration or anger, anxiety, difficulty concentrating, restlessness, increased appetite or weight gain, depressed or sad mood and insomnia. Anxiety and depression were assessed using the Beck Depression Inventory 47 and Kessler Psychological Distress Scale (K-10) 48. Physical and mental wellbeing was measured with the Short Form (SF)-36 49. Adverse events from the use of the patch and antidepressant were documented at 12 weeks using a checklist.

##### Ethical considerations

This research was approved by the Human Research Ethics Committees of the University of New South Wales, Justice Health NSW, the NSW Department of Corrective Services, the Aboriginal Health and Medical Research Council of NSW and the Queensland Corrective Services Research Committee. Written consent was required to participate. As part of this process, prisoners were informed that participation was voluntary and that they could withdraw from the study at any time without consequence. Inmates who experienced side effects during the course of the trial were referred to prison medical services for further assessment.

### Statistical analyses

Data were analysed using SAS version 9.2. For the smoking-related outcome variables, overall intention-to-treat analyses 44 were conducted (treatment group *n* = 206) versus control group (*n* = 219) together with subgroup analyses based on patterns of treatment and use of pharmacotherapies. For these analyses, missing data were classified either as continuing smoking or as not achieving a 50% reduction in tobacco use. Odds ratios (ORs) and associated 95% confidence intervals (CI) are also reported, with the control group as the reference point (OR = 1.00). The threshold for statistical significance was *P* ≤ 0.05.

## Results

### Flow of participants through the study

**Figure 1:**
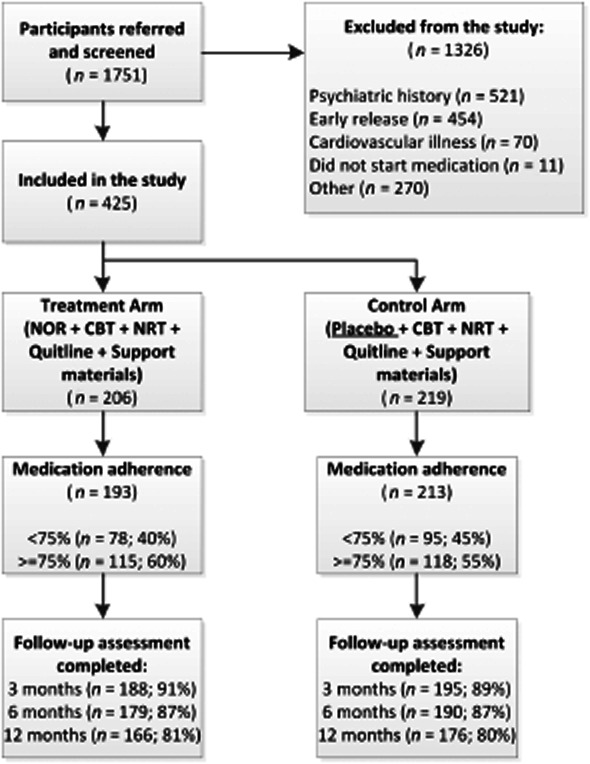
Flowchart of recruitment and retention of participants through the trial

Overall, 1751 prisoners expressed an interest in the study and were screened to determine suitability for the study (Fig. 1). Almost one-quarter (*n* = 425) fulfilled the inclusion criteria and were randomized to one of the two arms of the study: treatment (*n* = 206) or control (*n* = 219), and between 80 and 91% prisoners completed the scheduled follow-up at 3, 6 and 12 months (Fig. 1). Histories of psychiatric illness (including suicide and self-harm) and short sentences preventing follow-up were the most common reasons for excluding potential participants.

### Demographic, offending history and smoking characteristics

The treatment and control groups were similar in terms of their demographic, offending history and smoking characteristics, apart from a higher proportion of Aboriginal prisoners in the treatment arm (22.3 versus 8.2%, *P* < 0.01) (Table 2). The average age of the participants was 33.5 years, 15% were of Aboriginal origin and 75% were born in Australia. A high proportion (44%) of participants had left school with no qualification and the mean school-leaving age was 15 years. More than one-third of participants (38%) reported that they had been institutionalized as a child by either juvenile detention or out of home care. Nearly two-thirds (64%) of participants had been incarcerated previously; the median sentence length of participants was 3.6 years.

**Table 2:** Baseline demographics, offending history and smoking characteristics by treatment or control group

	Treatment	Control	Total
			
Characteristics	% (n = 206)	% (n = 219)	% (n = 425)
Demographic characteristics			
Mean age (years) (+SD; range)	32.8 (10.1; 18–65)	34.1 (10.3; 19–63)	33.5 (10.2; 18–65)
Aboriginal origin	22.3	8.2	15.1**
Born in Australia	73.2	76.4	74.9
Left school with no qualification	43.2	43.8	43.5
Mean age left school (+SD; range)	15.0 (2.3; 0–26)	15.2 (1.7; 11–22)	15.1 (2.0; 0–26)
Institutionalized as a child (juvenile detention and/or placed in care)	39.3	36.5	37.9
Homeless prior to prison	9.2	5.0	7.1
Employed while in prison	74.3	69.9	72.0
Offending history			
Previously incarcerated	64.1	63.5	63.8
Median number adult prison terms (+SD; range)	3.4 (3.1; 1–15)	3.2 (2.7; 1–20)	3.3 (2.9; 1–20)
Incarcerated 5+ years at baseline	17.0	19.2	18.1
Median years in prison at baseline (+SD; range)	1.9 (4.1; 0–23)	1.8 (4.3; 0–27)	1.9 (4.2; 0–27)
Sentence length 5+ years	34.5	33.8	34.1
Median sentence length in years (+SD; range)	3.6 (11.8; 0–112)	3.5 (16.1; 0–116)	3.6 (14.2; 0–116)
Smoking behaviours and history			
Mean age first smoked tobacco (+SD; range)	13.6 (4.2; 5–37)	13.8 (4.3; 6–39)	13.7 (4.3; 5–39)
Mean age first smoked tobacco daily (+SD; range)	15.4 (4.0; 5–37)	15.5 (4.3; 7–39)	15.5 (4.2; 5–39)
Mean years smoked tobacco (+SD; range)	19.2 (10.1; 0–52)	20.3 (10.4; 1–49)	19.8 (10.2; 1–52)
Mean carbon monoxide reading (+SD; range)	13.9 (7.2; 2–43)	14.6 (8.3; 1–59)	14.3 (7.8; 1–59)
Mean cigarettes smoked per day (+SD; range)	23.6 (10.3; 5–75)	22.7 (9.3; 3–75)	23.2 (9.8; 3–75)
Smoke 20+ cigarettes per day	69.4	70.8	70.1
High tobacco dependence (Fagerström 6+)	81.1	84.5	82.8
Share a cell with a smoker	37.6	29.7	33.5
Smoke White Ox (loose tobacco)	98.1	96.4	97.2
Smoking cessation history			
Mean times tried to quit smoking (+SD; range)	2.9 (9.2; 0–128)	2.3 (3.9; 0–50)	2.6 (7.9; 0–128)
Any type of quitting behaviour in past year	75.7	71.2	73.4
Very determined to cut down smoking	83.5	83.6	83.5
Very determined to quit smoking	87.9	86.8	87.3

** *P* < 0.01. SD: standard deviation.

The mean age at which participants first smoked tobacco was 13.7 years, with an average of 20 years of smoking and 23.2 cigarettes smoked per day. Most participants scored ≥6 on the FTND, suggesting high tobacco dependence. Almost three-quarters had tried to quit smoking in the past year (mean number of quit attempts 2.6).

### Multi-component intervention

#### Primary efficacy end-point

Based on an intention-to-treat analysis and a cut-point for CO of ≤10 p.p.m., continuous abstinence between the treatment and comparison groups was not statistically different at 3 months (23.8 versus 16.4%), 6 months (17.5 versus 12.3%) and 12 months (11.7 versus 11.9%) (Table 3). Similarly, there was no significant difference in point prevalence abstinence between the treatment and control groups at the scheduled follow-ups.

**Table 3:** Continuous and point-prevalence abstinence rates and smoking reduction status

	3 months (n = 383)			6 months (n = 369)			12 months (n = 342)		
			
Measures/Groups	%	Odds ratio	95% CI	%	Odds ratio	95% CI	%	Odds ratio	95% CI
Continuous abstinence									
Comparison group (*n* = 219)	16.4			12.3			11.9		
Treatment group (*n* = 206)	23.8	1.59	0.98–2.56	17.5	1.51	0.88–2.58	11.7	0.98	0.54–1.77
Point prevalence									
Comparison group (*n* = 219)	19.6			14.2			14.6		
Treatment group (*n* = 206)	27.7	1.57	1.00–2.46	19.4	1.46	0.87–2.44	12.1	0.81	0.46–1.42
Smoking reduction of 50% or greater relative to baseline									
Comparison group (*n* = 219)	88.8			77.4			77.4		
Treatment group (*n* = 206)	89.9	1.12	0.59–2.15	81.5	1.29	0.77–2.14	72.0	0.75	0.45–1.23

CI: confidence interval.

We calculated the abstinence rates in our study and found that the abstinence rates using a cut-point for CO of ≤10 p.p.m. and ≤5 p.p.m. were similar.

Using the ≤5 p.p.m. cut-off for continuous abstinence, the treatment and control groups were not statistically different at 3 months (22.8 versus 16.0%), 6 (17.5 versus 11.9%) and 12 months (11.7 versus 11.4%). Point prevalence abstinence, using the ≤5 p.p.m. cut-off between the treatment and control groups, was also not statistically different at 3 months (25.7 versus 18.7%), 6 (19.4 versus 13.7%) and 12 months (12.1 versus 14.2%).

## Discussion

This is the first RCT of a smoking cessation intervention among male prisoners. We compared a multi-component smoking cessation intervention including active NOR versus placebo over a 12-month period. Combination therapy using anti-smoking medications, antidepressants and NRT for smoking cessation have been shown to be more effective than NRT alone 29,31, although not consistently 50. In this study, we found no significant difference in an intention-to-treat analysis between the two study groups, suggesting that the additional use of NOR does not enhance quit rates for tobacco in the longer term. However, we found smoking cessation rates comparable to the community, including higher quit rates for participants on active NOR at 3 months (23.8 versus 16.4%) and 6 months (17.5 versus 12.3%), but very similar at 12 months (11.7 versus 11.9%). Considering the stresses associated with being in prison, these abstinence rates are very encouraging and indicate that offering smoking cessation interventions to prison inmates is worthwhile.

These findings are encouraging, as prisoners have long and entrenched smoking histories and they smoke pouch tobacco, which has high levels of nicotine and tar which puts them at increased risk for cancer, heart disease and chronic obstructive pulmonary disease 51,52. We have reported multiple cardiovascular risk factors previously in this group 53. Mortality studies of prisoners have also found increased death rates from cardiovascular conditions compared with the community 54.

The majority of participants in both groups reduced their smoking by at least half relative to baseline levels, including abstaining at all three follow-up points. Smokers who reduced their tobacco use significantly can maintain reductions for longer periods, which may assist future cessation efforts suggesting that reduction *per se* is also a worthwhile outcome to pursue 43,55. Gradual reduction before a planned attempt at stopping smoking may increase eventual success at quitting 56, especially if nicotine dependence is lessened 57.

As this trial tested a multi-component smoking cessation intervention consisting of evidence-based components, we are unable to identify which element within the package had the most impact on the study findings. As the pharmacotherapy was supervised at the prison clinic on a daily basis, this was likely to lead to better compliance than may be found in community-based studies. Non-compliance with NRT has been associated with high nicotine dependence and low motivation to quit 58–60. Results for use of the combination medications (68%) are similar to those of a UK study (78%) using combination NRT and NOR 50. In future studies, other pharmacotherapy regimens to assist with smoking cessation should be considered, such as combination NRT.

### Comparison with other studies

The only other published prisoner smoking cessation intervention study 26 compared women in one US prison, and reported that point prevalence abstinence in the treatment group at 6 and 12 months was 14 and 12% 26,27; we found 19 and 12% at 6 and 12 months. Our point prevalence abstinence rates among prisoners are remarkably similar to a number of community studies, where approximately 12% remained abstinent at 12 months 61,62, suggesting that prisoners can quit at similar rates to the community despite living in a culture in which tobacco is deeply entrenched and even encouraged 63. By contrast, community groups generally experience a strong anti-smoking environment with a variety of bans and restrictions on tobacco smoking in public places. Conversely, prisons are characterized as having a strong pro-smoking culture 64.

### Strengths and limitations of the study

Recruitment and follow-up was undertaken in most of the 29 prisons in one state (NSW), thus limiting the possibility of selection bias on the basis of prisoner group at the different facilities (e.g. prisoners housing younger offenders, sex offender, low-security prisoners). This is endorsed by the demographic and smoking characteristics of the sample which, apart from containing fewer Aboriginal prisoners, was similar to the overall prisoner population in New South Wales 7. The results are therefore likely to be generalizable to other jurisdictions both within Australia and internationally.

The study was conducted with a pre-specified study protocol and analysis plan and *a priori* specification of end-points. We conducted our study in line with Consolidated Standards of Reporting Trials (CONSORT) guidelines 64, and adhered to the standard established by West in considering all participants lost to follow-up as continuing smokers 65.

The major strength of our study was the blinding and the use of an objective, biochemical measure to determine abstinence as recommended by the Society for Research on Nicotine and Tobacco (SRNT) working group 66. We found the biochemical verification to be consistent with self-reported abstinence. Follow-up rates were high. Contrary to the perception of the prisoner population as a ‘captive audience’, this group is highly mobile, with 52% of those enrolled in the study transferred to another prison for a range of reasons. One hundred participants were released into the community, of whom 56% were successfully followed-up.

We excluded 30% of potential participants as they had fewer than 6 months of their prison sentence remaining and would not be available for follow-up. Few participants accessed the Quitline to support their quit attempt, suggesting that this component of the intervention had little impact on the outcome. Many participants expressed a desire to receive more than two sessions of CBT, which may have increased the quit rate. Further studies offering more intense CBT are recommended.

Other agents (e.g. varenicline) became available during the development of the study and it is possible that use of this anti-smoking medication may have yielded different results. We selected NOR on the basis of its preferable side-effect profile and the lower demand on the prison medical services (requiring once-daily administration). We were also cognisant of the cost of NOR, which is off-patent, versus other smoking cessation drugs, and surmised that, when in the community, this population are more likely to use low-cost alternatives.

## Conclusions

Our study set out to determine the effectiveness of a multi-component intervention with the addition of active NOR for prisoners, who are rarely the focus of health promotion campaigns to assist smoking cessation.

The main findings are:

1. Adding NOR to the multi-component intervention including NRT did not result in any significant differences in smoking cessation at 3-, 6- and 12-month follow-up; and
2. Quit smoking rates were only marginally lower than those observed in community studies.

There is an urgent need for the development of effective smoking cessation interventions for those prisoners who did not respond to the brief multi-component intervention offered in this study. Research is recommended to evaluate the effect of a smoking cessation intervention with extended CBT, longer-term NRT and other anti-smoking medications. The development of culturally appropriate interventions for Aboriginal and other cultural groups also needs further consideration. As a harm reduction strategy for those prisoners who only want to reduce their tobacco consumption, specific interventions focusing on reduction rather than smoking interventions need to be developed 55,67. However, we advocate reduction as a means to eventual quitting.

Further research with longer-term follow-up is necessary to reinforce non-smoking in the prison system, where smoking rates are high and the prevailing culture promotes tobacco use.

## Clinical trial registration

This trial is registered with the Australian New Zealand Clinical Trials Registry #12606000229572. http://www.anzctr.org.au/trial_view.aspx?ID=1329
